# IgG antibody response to SARS-CoV-2 infection and its influencing factors in lymphoma patients

**DOI:** 10.1186/s12865-024-00596-1

**Published:** 2024-01-13

**Authors:** Huan Xie, Jing Zhang, Ran Luo, Yan Qi, Yizhang Lin, Changhao Han, Xi Li, Dongfeng Zeng

**Affiliations:** Department of Hematology, Daping Hospital, Army Medical University, No. 10, Daping Changjiang Branch Road, Yuzhong District, Chongqing, 400042 China

**Keywords:** Lymphoma, COVID-19, SARS-CoV-2, Antibody, CD20

## Abstract

**Background:**

The ability of generating effective humoral immune responses to SARS-CoV-2 infection has not been clarified in lymphoma patients. The study aimed to investigate the antibody (Ab) production after SARS-Cov-2 infection and clarify the factors affecting the Ab generation in these patients.

**Patients & methods:**

80 lymphoma patients and 51 healthy controls were included in this prospective observational study. Clinical factors and treatment regimens affecting Ab positive rate (APR) and Ab levels were analyzed by univariate and multivariate methods.

**Results:**

The anti-SARS-CoV-2 IgG APR and Ab levels in lymphoma patients were significantly lower than those in healthy controls. Lymphoma patients with COVID-19 vaccination had significantly higher APR and Ab levels compared with those without vaccination. Additionally, the use of dexamethasone for COVID-19 treatment had a negative impact on Ab levels. For the impact of treatment regimens on the APR and Ab levels, the results showed that patients treated with ≥ 6 times CD20 monoclonal Ab (mAb) and patients treated with autologous hematopoietic stem cell transplantation (ASCT) prior to infection produced a statistically lower APR and Ab levels compared with those treated with 1–5 times CD20 mAb and those treated without ASCT, respectively. Furthermore, multiple regression analysis indicated that the number of anti-CD20 treatment was an independent predictor for both APR and Ab levels.

**Conclusions:**

Humoral immune response to SARS-CoV-2 infection was impaired in lymphoma patients partly due to anti-CD20 and ASCT treatment. COVID-19 vaccination may be more needed for these patients.

**Supplementary Information:**

The online version contains supplementary material available at 10.1186/s12865-024-00596-1.

## Introduction

Although various measures had been taken globally to address highly transmissible SARS-CoV-2 variants, the Omicron wave swept across China in 2022 [1]. Particularly, the SARS-CoV-2 outbreak increased the risk of death in patients with malignant hematological diseases. Among them, the mortality rate of hospitalized patients was as high as 31.2% [2]. Furthermore, the lymphoma patients are more susceptible to coronavirus disease 2019 (COVID-19). In addition to the pathogenesis of malignant cloning of immune cells, the anti-tumor regimens, such as monoclonal antibodies, pathway inhibitors, and autologous hematopoietic stem cell transplantation (ASCT), also exerted harmful effects on the immune system [3].

The dynamics of specific antibody (Ab) levels following SARS-CoV-2 infection in healthy populations have been well documented and confirmed by a large body of research data [4, 5]. Accordingly, Ab levels reached the initial peak at around one month after infection, and then gradually decreased into the plateau period [6–8]. Maintaining Ab levels might be an effective mean of preventing reinfection or reducing the incidence of severe cases. In contrast, lymphoma patients often exhibited an adaptive humoral immune deficiency, and their Ab response to SARS-CoV-2 was usually not ideal [9, 10]. Meanwhile, the studies about the immune response to SARS-CoV-2 in lymphoma patients mainly focused on the immune response after vaccination (i.e., inactivated virus) [3, 10, 11]. For example, Chang et al. found that the anti-CD20 treatment and the number of circulating B lymphocytes strongly predicted the vaccine response [11]. Nevertheless, data from studies on the ability of lymphoma patients to produce specific Ab after SARS-CoV-2 infection and the factors that influence this ability, particularly against Omicron, remain limited.

CD20 is a surface protein of B cells that is expressed from pre-B cells to mature B cells, making it an important target for B-cell lymphomas [12]. A growing body of evidence suggested that the application of CD20 monoclonal Ab (mAb) was one of the main causes of humoral immunodeficiency in lymphoma patients [13–16]. Concretely, long-term use of the drug depleted mature B lymphocytes along with secondary hypogammaglobulinemia and weakened the humoral immune response to new pathogens in lymphoma patients. This not only increased the complications of infection, but also significantly reduced the ability of producing specific Ab and Ab titers following viral infection. All of these could increase the risk of reinfection with the virus. Ultimately, it might affect the long-term prognosis of lymphoma patients who survived during acute infection with SARS-CoV-2.

Currently, some researchers have demonstrated the impact of anti-CD20 treatment on the production of anti-SARS-CoV-2 IgG Ab [9, 16]. However, further research is needed regarding the relationship between anti-SARS-CoV-2 Ab levels and the clinical characteristics, as well as the details of treatment regimens in lymphoma patients. To elucidate these points, the present study conducted a prospective study on 80 Chinese lymphoma patients and 51 healthy controls infected with COVID-19. Here, the data of anti-SARS-CoV-2 IgG Ab positivity rate (APR) and Ab levels about two months after infection in those two groups were reported. More importantly, we analyzed the factors influencing APR and Ab levels and followed up the clinical outcome of the patients.

## Patients and methods

### Patients and healthy controls

This was a prospective observational study with longitudinal follow-up of lymphoma patients infected with COVID-19. Those participants were recruited from December, 2022 to January, 2023, and the follow-up period was up to December, 2023. This study was performed by the Hematology Department of Daping Hospital Affiliated to the Army Medical University. Inclusion criteria: patients who were diagnosed with lymphoma and received formal treatment before December, 2022; Lymphoma patients who survived after the acute phase of COVID-19 infection. Exclusion criteria: patients who had no history of COVID-19 infection; patients with non-treated lymphoma or diagnosed after COVID-19 infection. COVID-19 infection was confirmed by nucleic acid or antigen testing. Anti-SARS-CoV-2 IgG Ab levels were tested about two months (50–70 days) after the positive record of the virus.

Patients’ demographic and clinical data were collected from medical recordings including age, gender, vaccination history, diagnosis, disease stage, time of COVID-19 infection, severity of COVID-19, lymphocyte subsets, treatment regimen, and therapeutic efficacy. The severity of COVID-19 infection was classified (mild, moderate, severe, and critical) according to the Guideline for Coronavirus Disease [17]. 51 individuals without hematological and other chronic underlying diseases were simultaneously recruited as the healthy controls. The controls lived in the same city and were diagnosed with COVID-19 at the same period as the lymphoma group.

### Anti-SARS-CoV-2 IgG Ab detection

Peripheral blood samples were collected from both the lymphoma patients and healthy controls at about two months after COVID-19 infection. Data from participants two months after infection were analyzed because this period was about the plateau of the initial humoral immune response against SARS-CoV-2 and Ab concentrations were largely maintained at a relatively stable high level [4]. Following the procedure of 2019-nCoV IgG Ab test kit (Maccura, Cat.20203400496, Chengdu, China), the levels of IgG Ab to the SARS-CoV-2 total proteins were measured using magnetic particle-based chemiluminescence enzyme immunoassay (CLEIA) [18]. The sensitivity and specificity of the assay were 87.78% (95% CI: 83.95% ~ 90.80%) and 99.01% (95% CI: 97.71% ~ 99.58%), respectively. The cut-off value for anti-SARS-CoV-2 IgG Ab positivity and negativity was 0.999 S/CO. All IgG Ab detection and analysis were carried out in the same machine (Michael i3000 automatic chemiluminescence immune analyzer) in the hospital.

### Statistical analysis

The data of clinical characteristics and outcomes of the patients were collected. T-test or F-test were applied to compare the impact of different clinical characteristics and treatment regimens on the continuous values of anti-SARS-CoV-2 IgG Ab levels. The chi-square (χ^2^) test was used to compare the impact of different clinical characteristics and treatment regimens on the categorical variable, i.e., anti-SARS-CoV-2 IgG Ab positivity rate (APR). APR is defined as the percentage of the population with IgG Ab values greater than 0.999 S/CO [19]. A two-sided *P* value < 0.05 was considered to be statistically significant. Variables with *P* value < 0.05 in the univariate analysis were entered into the final multiple regression as independent variables. Multiple linear regression and binary logistic regression were used to perform multifactorial analyses of SARS-CoV-2 IgG Ab levels and APR, respectively.

## Results

### Clinical characteristics of patients and healthy controls

A total of 80 lymphoma patients (37 DLBCL, 8 MZL, 6 MCL, 5 FL, 7 HL, 13 TCL, and 4 other types of lymphoma) and 51 healthy controls with SARS-CoV-2 infection were enrolled. The clinical characteristics of all patients are shown in Table 1. The clinical characteristics of each patient were shown in the supplementary materials (https://www.jianguoyun.com/p/DcFlZ2MQsJ6gDBjxhq8FIAA). The median age of the lymphoma cohort was 58 years (range: 18 ~ 85 years) and 41 of the patients were male. The healthy controls included 15 males with a median age of 32 years (range: 15 ~ 46 years). All healthy controls were vaccinated with COVID-19, while only 56 (70.0%) of the lymphoma patient group were vaccinated. 70 patients (87.5%) received anti-lymphoma therapy within one year prior to COVID-19 infection, while the remaining 10 ended treatment due to achieving complete remission. 58 patients (72.5%) were treated with CD20 mAb (rituximab or ortuzumab), among whom 47 patients were combined with chemotherapy, 2 patients were combined with bruton tyrosine kinase inhibitor (BTKi), and 9 patients were combined with all three treatments. 12 patients (15%) were treated with BTKi, and 12 patients (15%) received ASCT prior to COVID-19 infection.

**Table 1:** Clinical characteristics of lymphoma patients (*n* = 80)

**Median age (range)**	58 (18 ~ 85)		**Received Treatment**	
**Male/Female**		41/39	Anti-CD20 + Chemo	47
**Diagnosis**			Anti-CD20 + BTKi	2
DLBCL		37	Anti-CD20 + Chemo + BTKi	9
TCL		13	BTKi alone	1
MZL		8	ASCT	12
HL		7	**Severity of COVID-19**	
MCL		6	Mild	57
FL		5	Moderate	16
OTL		4	Severe/Critical	7
**Risk stratification (NCCN-IPI)**			**Treatment of COVID-19**	
Low		26	Symptomatic treatment	57
Moderate		25	Oxygen therapy/steroids/antiviral	23
High		29	mAb/convalescent plasma	3
**Vaccination history**		56	ICU admission	2

**Abbreviations**: DLBCL, Diffuse large B-cell lymphoma; TCL, T-cell lymphoma; MZL, Marginal zone lymphoma; HL, Hodgkin’s lymphoma; MCL, mantle cell lymphoma; FL, Follicular lymphoma; OTL, Other types of lymphoma; NCCN-IPI, National Comprehensive Cancer Network International Prognostic Index; Chemo, chemotherapy; ICU, intensive care unit

57 lymphoma patients (71.3%) and all 51 healthy controls with mild COVID-19 received only symptomatic treatment, such as antipyretic and antitussive treatment. 23 patients (28.8%) received oxygen therapy, among whom 19 patients (23.8%) were treated with antiviral drugs, and 9 patients (11.3%) were treated with dexamethasone. 3 patients (3.8%) received mAb or convalescent plasma therapy. 2 patients (2.5%) required mechanical ventilation in the ICU.

### Humoral response of lymphoma patients to SARS-CoV-2

**Fig. 1:**
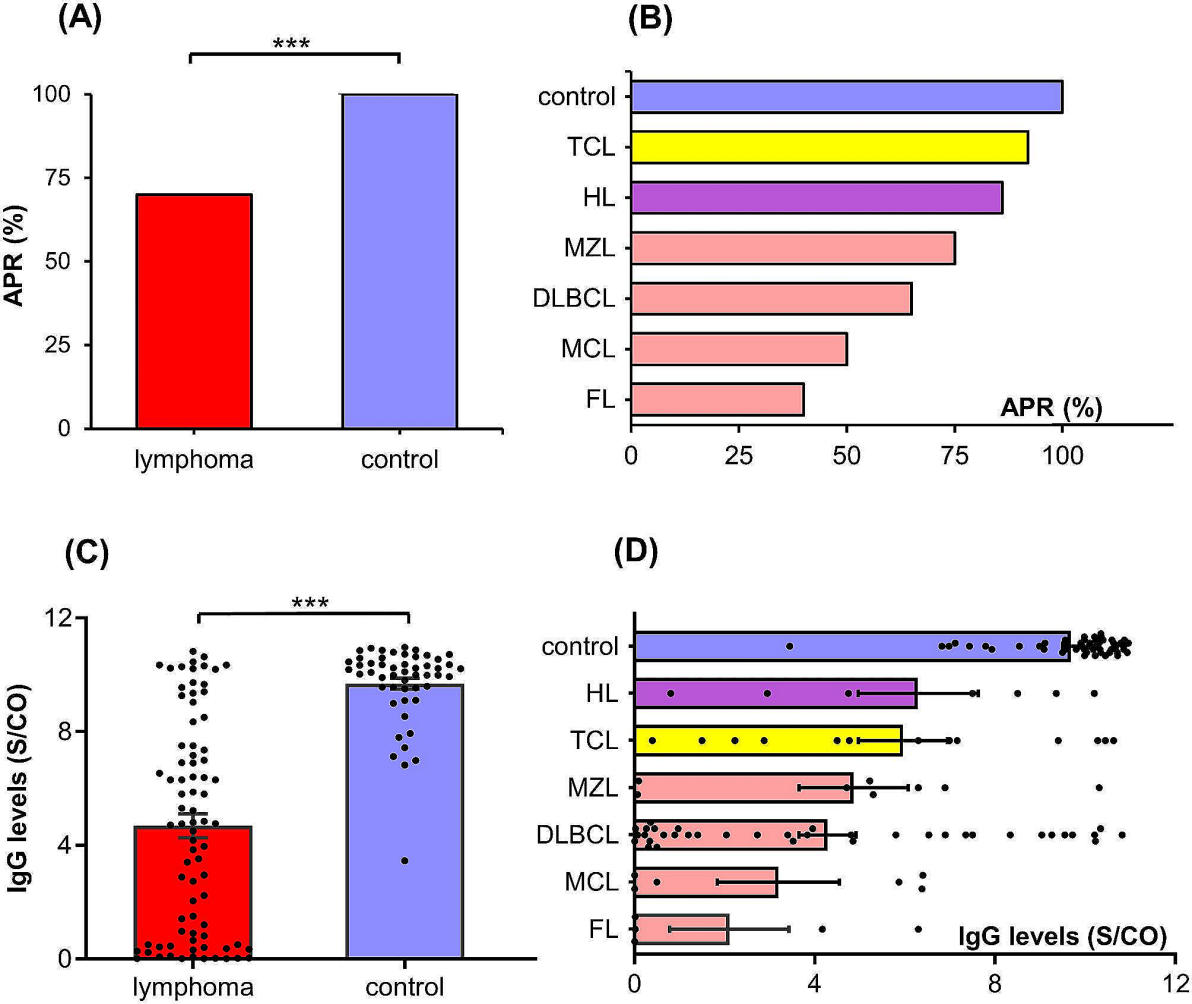
The comparison of anti-SARS-CoV-2 IgG APR and Ab levels between lymphoma patients and healthy controls (**A** and **C**). The anti-SARS-CoV-2 IgG APR and Ab levels among each type of lymphoma and healthy controls (**B** and **D**)

The anti-SARS-CoV-2 IgG APR and Ab levels in lymphoma patients and healthy controls are exhibited in Fig. 1. The χ^2^ test and t-test test revealed that the IgG APR and average Ab levels in lymphoma patients were significantly lower than those in healthy controls (70% vs. 100%, *P* < 0.001; 4.69 vs. 9.69 S/CO, *P* < 0.001, see Fig. 1A and **C**). Based on the classification of lymphoma, the subtypes of lymphoma with low to high IgG APR were FL (40%), MCL (50%), DLBCL (65%), MZL (75%), HL (86%), and TCL (92%), respectively (see Fig. 1B), and Ab levels were FL (2.10 S/CO), MCL (3.20 S/CO), DLBCL (4.28 S/CO), MZL (4.86 S/CO), TCL (5.96 S/CO), and HL (6.30 S/CO), respectively (see Fig. 1D). Compared with the healthy controls, the IgG APR (Ps < 0.05) and Ab levels (Ps < 0.004) in each subgroup were significantly decreased.

### Factors affecting humoral response in lymphoma patients

#### Effects of clinical characteristics on anti-SARS-CoV-2 IgG APR and Ab levels

We first analyzed the impact of age, gender, vaccination history, lymphoma staging, disease status before COVID-19 infection, severity of COVID-19, and the use of dexamethasone for COVID-19 treatment on anti-SARS-CoV-2 IgG APR and Ab levels (see Table 2). The results found that vaccinated lymphoma patients had significantly higher IgG APR (76.8% vs. 54.2%, *P* = 0.04) and Ab levels (5.63 vs. 2.48 S/CO, *P* < 0.001) than unvaccinated patients. Regardless of whether the patients were vaccinated, the IgG APR (Ps < 0.001) and Ab levels (Ps < 0.001) of the two groups were significantly lower than those of the healthy controls. Additionally, the use of dexamethasone for COVID-19 treatment had a negative impact on Ab levels (2.22 vs. 5.00 S/CO, *P* = 0.004). Age, gender, lymphoma staging, disease status, and severity of COVID-19 had no significant effects on both APR and Ab levels (Ps > 0.06).

**Table 2:** The influence of clinical factors on the IgG APR and Ab levels against SARS-CoV-2

	IgG APR	*P*-value	Mean (SD) IgG levels (S/CO)	*P*-value
Elderly group (≥ 60 years)	71.0%	0.88	4.54 (3.57)	0.78
Young group (< 60years)	69.4%		4.78 (3.86)	
Male	65.9%	0.41	4.52 (3.87)	0.80
Female	74.4%		4.86 (3.62)	
Vaccination group	76.8%	**0.04**	5.63 (3.83)	< **0.001**
Unvaccinated group	54.2%		2.48 (2.32)	
Stage I-II lymphoma	81.0%	0.20	5.69 (3.91)	0.17
Stage III-IV lymphoma	66.1%		4.33 (3.63)	
CR/PR/SD group	71.0%	0.62	4.85 (3.70)	0.35
PD group	63.6%		3.63 (3.89)	
Mild COVID-19	71.9%	0.55	5.16 (3.80)	0.06
Moderate/Severe/Critical COVID-19	65.2%		3.51 (3.33)	
Treatment for COVID-19 with dexamethasone	55.6%	0.32	2.22 (2.06)	**0.004**
Treatment for COVID-19 without dexamethasone	71.8%		5.00 (3.78)	
Control group	100%	--	9.69 (1.38)	--

**Abbreviations**: CR, complete response; PR, partial response; SD, stable diseases; PD, progressive disease

**Fig. 2:**
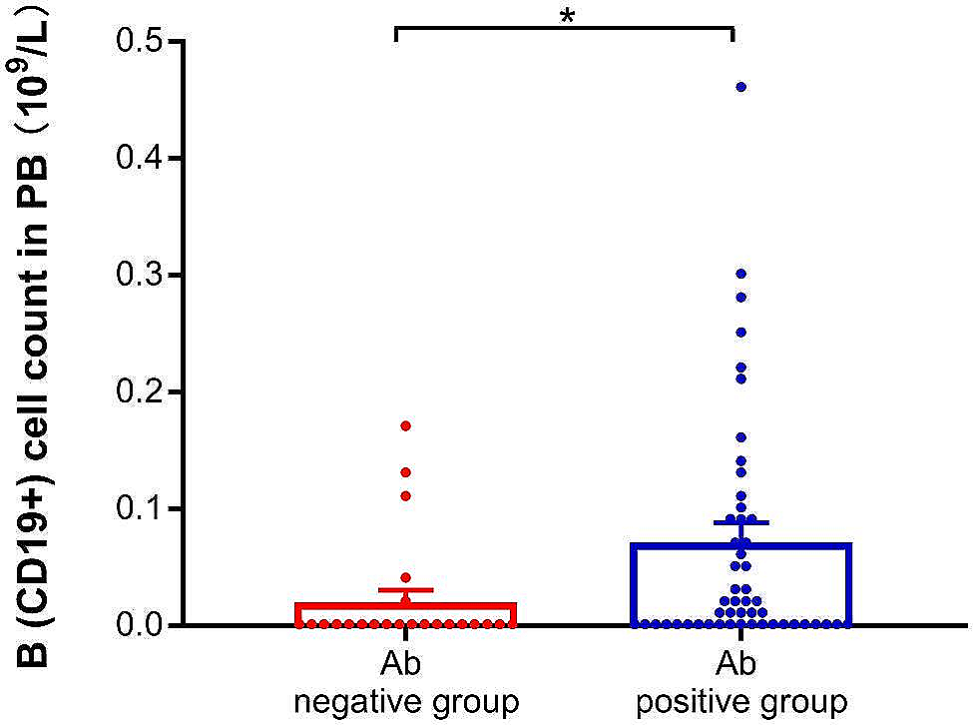
The comparison of B (CD19+) cell counts in peripheral blood (PB) between anti-SARS-CoV-2 IgG Ab negative group and positive group

Meanwhile, the information of 75 lymphoma patients’ lymphocyte subsets was collected (five patients were not tested) two months after COVID-19 infection. The patients were divided into anti-SARS-CoV-2 IgG Ab positive group (*n* = 52) and negative group (*n* = 23). The results showed that the absolute value of B lymphocytes in the IgG positive group was significantly higher than that in the negative group (0.0715 vs. 0.0204 × 10^9^/L, *P* = 0.01) (see Fig. 2), while there were no significant differences in CD4 + T, CD8 + T and NK cells between the two groups (Ps > 0.38).

#### Effect of treatment on anti-SARS-CoV-2 IgG APR and Ab levels

##### Anti-CD20 treatment

**Fig. 3:**
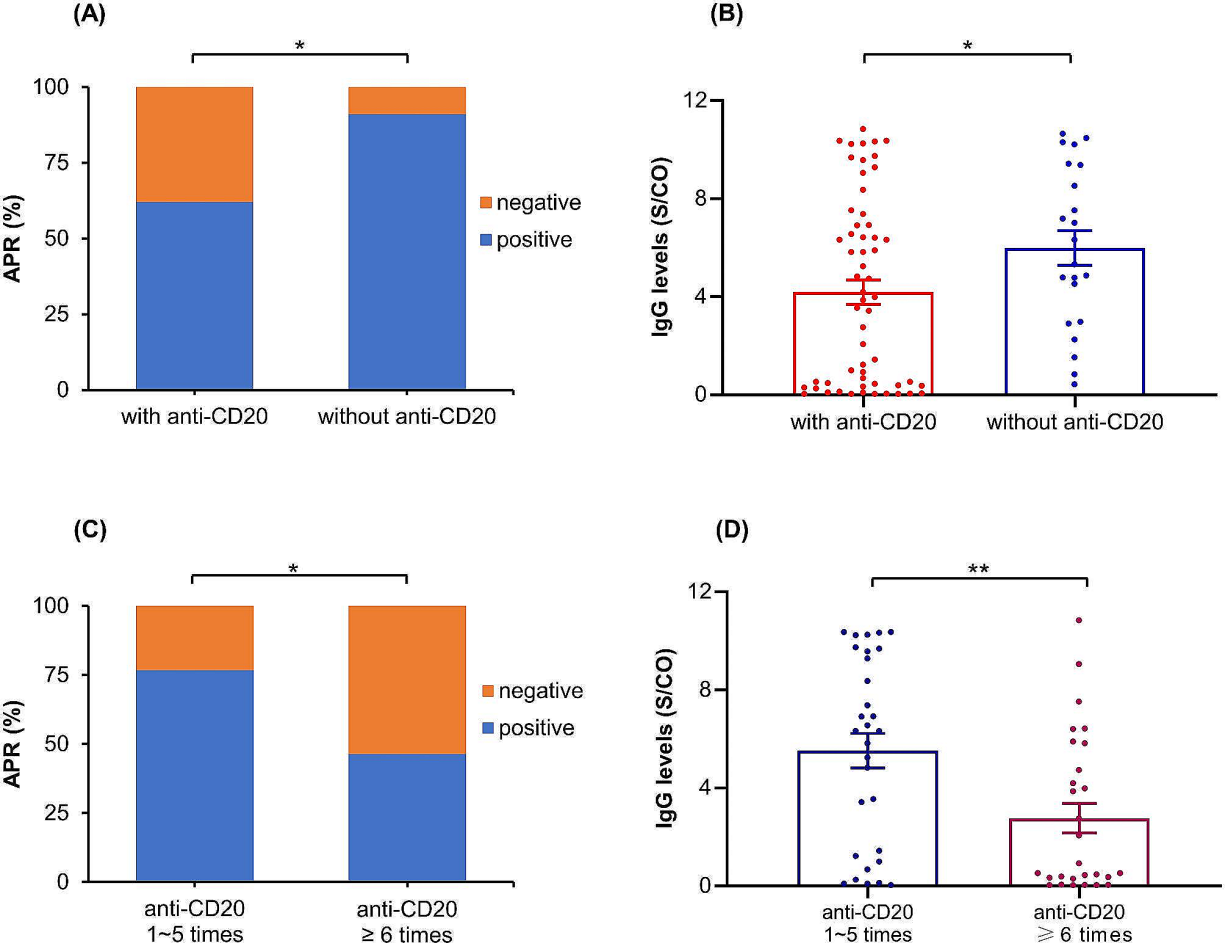
The comparison of anti-SARS-CoV-2 IgG APR and Ab levels in lymphoma patients treated with and without anti-CD20 (**A** and **B**). The comparison of anti-SARS-CoV-2 IgG APR and Ab levels based on the number of anti-CD20 (**C** and **D**)

In this study, 58 patients underwent anti-CD20 treatment, including 77.6% aggressive and 20.7% indolent B-cell lymphoma. The results showed that the anti-SARS-CoV-2 IgG APR and Ab levels were significantly lower in patients who were previously received anti-CD20 treatment than those in patients who were not received it two months after COVID-19 infection (62.1% vs. 90.9%, *P* = 0.01; 4.19 vs. 5.99 S/CO, *P* = 0.04) (see Fig. 3A and B).

Then, we analyzed the Ab production ability between the subgroup with their last anti-CD20 treatment within 3 months prior to infection and the subgroup with their last anti-CD20 treatment more than 3 months prior to infection. The results revealed no significant differences on APR (56.1% vs. 76.5%, *P* = 0.15) and IgG levels (4.22 vs. 4.12 S/CO, *P* = 0.92). Next, the impact of the times of receiving CD20 mAbs treatment on the IgG APR and Ab levels of patients was also analyzed. The results showed that there were no significant differences on both when the boundary was 4 times (58.1% vs. 73.3%, *P* = 0.30; 3.66 vs. 5.71 S/CO, *P* = 0.07). There was only a significant difference in Ab levels when the boundary was 5 times (52.8% vs. 77.3%, *P* = 0.06; 3.16 vs. 5.88 S/CO, *P* = 0.007). Furthermore, the IgG APR and Ab levels were significantly lower in patients who received ≥ 6 times CD20 mAbs than those who were treated 1 ~ 5 times CD20 mAbs (46.4% vs. 76.7%, *P* = 0.02; 2.76 vs. 5.52 S/CO, *P* = 0.004) (see Fig. 3C and D). Additionally, among these 58 patients, 23, 9, 8, and 7 patients were treated with anti-CD20 Ab combined with CHOP-like, Bendamustine, Gemox, and MTX regimens within one year prior to infection, respectively. There were no significant differences among the four subgroups on IgG levels (5.94 vs. 4.44 vs. 4.13 vs. 2.56, Ps > 0.08).

##### BTKi treatment

**Fig. 4:**
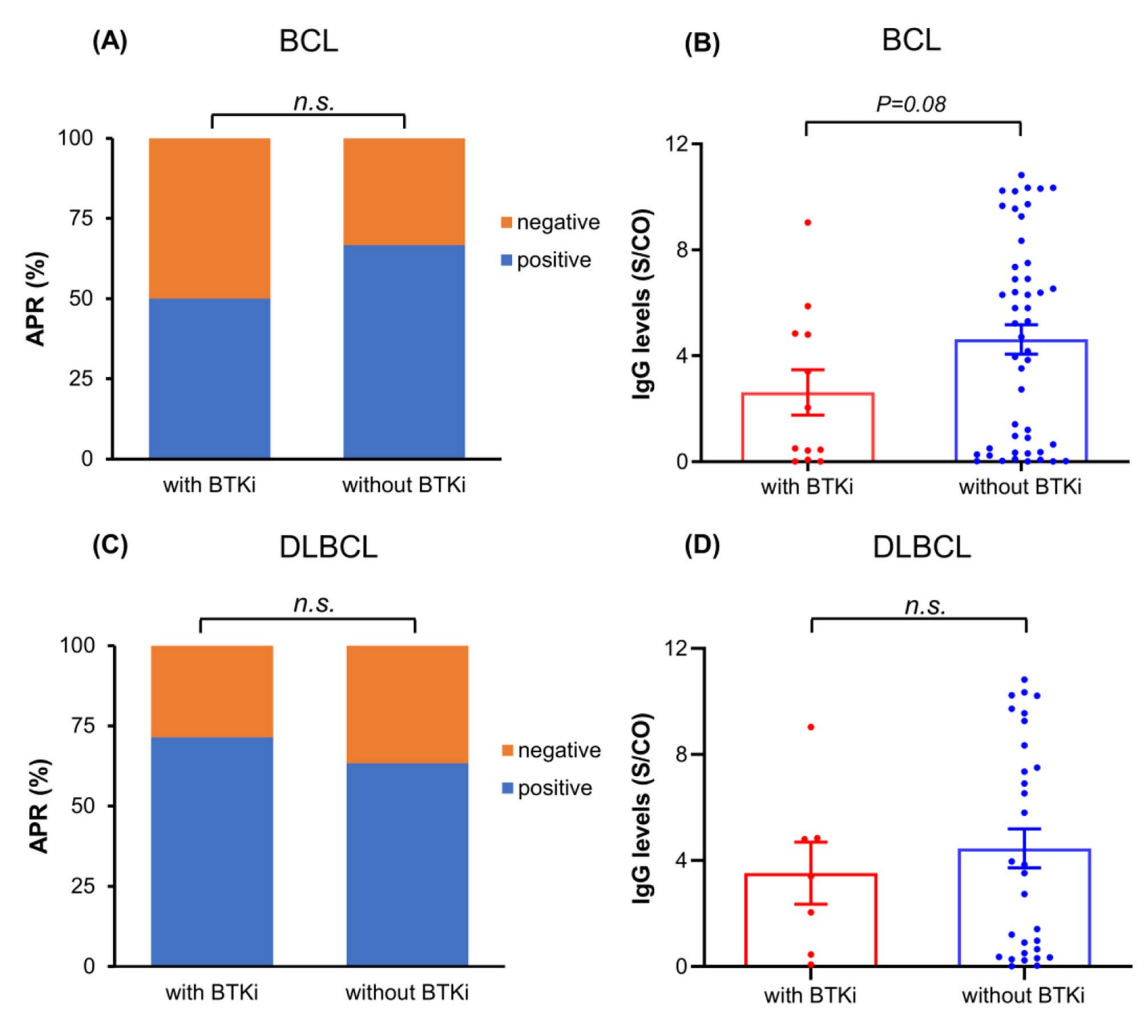
The comparison of anti-SARS-CoV-2 IgG APR and Ab levels in BCL (**A** and **B**) and DLBCL (**C** and **D**) patients treated with and without BTKi

A total of 12 patients (15.0%) received BTKi treatment, including 7 DLBCL, 4 MCL, and 1 VM, accounting for 20% of B-cell lymphoma (BCL). Further nonparametric Mann-Whitney test showed that the IgG Ab level in BCL patients who were previously treated with BTKi was slightly lower than that in patients who were not treated with BTKi two months after infection (2.62 vs. 4.62 S/CO, *P* = 0.08). However, there was no significant difference in APR between the two groups (50% vs. 66.7%, *P* = 0.28) (see Fig. 4A and B). In addition, oral BTKi had no significant effects in APR (71.4% vs. 63.3%, *P* = 0.69) and IgG levels (3.52 vs. 4.46 S/CO, *P* = 0.51) among DLBCL patients (see Fig. 4C and D).

##### ASCT treatment

**Fig. 5:**
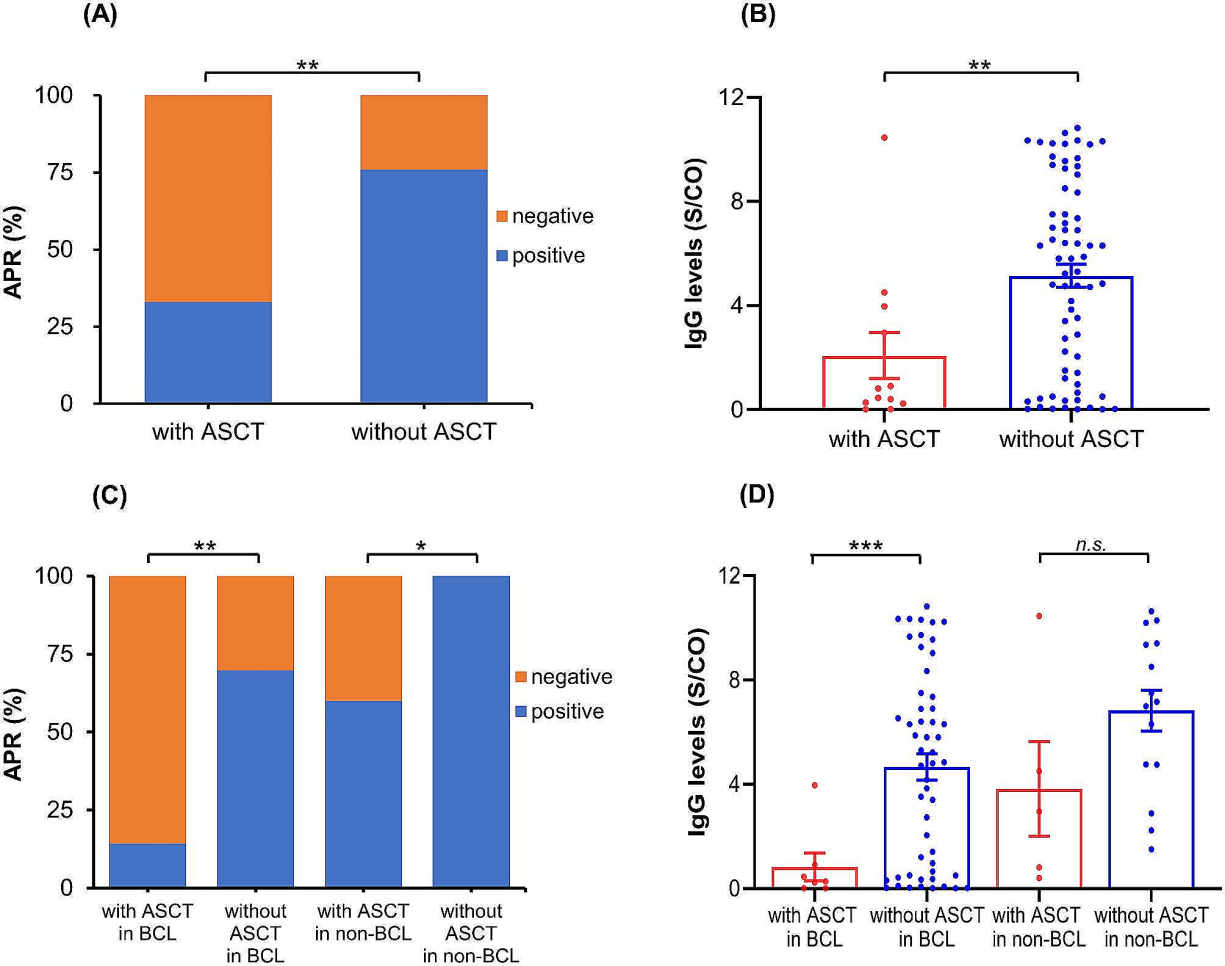
The comparison of anti-SARS-CoV-2 IgG APR and Ab levels in lymphoma patients treated with and without ASCT therapy (**A** and **B**). The comparison of anti-SARS-CoV-2 IgG APR and Ab levels in BCL and non-BCL subtypes treated with and without ASCT (**C** and **D**)

A total of 12 patients (15.0%) received ASCT therapy, including 5 DLBCL, 1 MCL, 1 FL, 2 HL, and 3 TCL. Detailed analysis showed that the anti-SARS-CoV-2 IgG APR and Ab levels of patients treated with ASCT were significantly lower than those of patients treated without ASCT (33.3% vs. 76.5%, *P* = 0.003; 2.08 vs. 5.15 S/CO, *P* = 0.007) (see Fig. 5A and B). In addition, the time interval between transplantation and infection did not significantly correlate to the Ab levels in patients who received ASCT therapy (*r* = 0.15, *P* = 0.64). Lymphoma patients were further divided into BCL and non-BCL subgroups. The results showed that, in the BCL subgroup, the IgG APR and Ab levels in the ASCT group were significantly lower than those in the non-ASCT group (14.3% vs. 69.8%, *P* = 0.004; 0.83 vs. 4.67 S/CO, *P* < 0.001), whereas in non-BCL subgroup, there was a significant difference between ASCT group and non-ASCT group in APR (60.0% vs. 100.0%, *P* = 0.01), but not in Ab levels (3.82 vs. 6.83 S/CO, *P* = 0.18) (see Fig. 5C and D).

#### Multiple regression analysis on anti-SARS-CoV-2 IgG APR and Ab levels

We further performed the multiple regression analyses on anti-SARS-CoV-2 IgG APR and Ab levels, taking the number of anti-CD20 treatment, ASCT, the absolute value of B lymphocytes, vaccination history, and treatment for COVID-19 with dexamethasone as independent variables. The regression analysis confirmed that the number of anti-CD20 treatment (Exp(B) = 0.795 [CI: 0.669 ~ 0.946], *P* = 0.009) and ASCT (Exp(B) = 0.057 [CI: 0.007 ~ 0.445], *P* = 0.006) were independent predictors on anti-SARS-CoV-2 IgG APR. Furthermore, the number of anti-CD20 treatment was an independent predictor on anti-SARS-CoV-2 IgG Ab levels (B = -0.232 [CI: -0.414 ~ -0.051], *P* = 0.01) (see Table 3).

**Table 3:** Multiple regression analysis results on anti-SARS-CoV-2 IgG APR and Ab levels

Variables	anti-SARS-CoV-2 IgG APR		anti-SARS-CoV-2 IgG Ab levels	
	Exp (B) (95% confidence interval)	*P*-value	B value (95% confidence interval)	*P*-value
Anti-CD20 times	0.795 (0.669 ~ 0.946)	**0.009**	-0.232 (-0.414 ~ -0.051)	**0.01**
ASCT	0.057 (0.007 ~ 0.445)	**0.006**	-1.746 (-3.998 ~ 0.505)	0.13
B (CD19+) cell count (10^3^/µL)	1.000013 (0.999998 ~ 1.000028)	0.09	-0.547 × 10^− 6^ (-8.851 × 10^− 6^ ~ 7.757 × 10^− 6^)	0.90
vaccination history	0.993 (0.190 ~ 5.175)	0.99	1.125 (-0.957 ~ 3.207)	0.29
Treatment for COVID-19 with dexamethasone	0.954 (0.133 ~ 6.873)	0.96	-2.005 (-4.557 ~ 0.546)	0.12

### Follow-up of clinical outcome

Finally, we followed up the clinical outcomes of the 80 lymphoma patients one year after infection. 33 patients (41.3%) continued to receive anti-lymphoma treatment and had progression-free survival, among whom 12 patients subsequently received ASCT. 21 patients (26.3%) stopped receiving treatment and had progression-free survival. 17 patients (21.3%) experienced disease progression, among whom 9 patients died due to disease progression. In addition, there were 2 deaths, one died of severe pneumonia caused by COVID-19 reinfection, and one died of severe peripheral neuropathy. 7 patients (8.8%) failed to be followed up. Further logistic regression analysis revealed the SARS-CoV-2 IgG levels did not significantly correlate with the clinical outcomes (Exp(B) = 0.96 [CI: 0.84 ~ 1.11], *P* = 0.61).

## Discussion

In the prospective study, we investigated the ability of producing anti-SARS-CoV-2 IgG Ab in 80 lymphoma patients after two-month COVID-19 infection and further analyzed the factors influencing the Ab levels. The results revealed that the Ab levels were significantly decreased in lymphoma patients compared with that in healthy controls. During the initial response, B cells are activated and terminally differentiate into long-lived plasma cells (LLPCs). The specific Abs secreted by LLPCs can be maintained for months or even years [20, 21]. Thus, the core of protective humoral immunity is precisely the production ability of LLPCs [21]. However, the above ability in lymphoma patients was defective, which reduced the production and maintenance ability of SARS-CoV-2 specific Abs in those patients [9, 10, 16]. Therefore, lymphoma patients may be the hardest hit by infection following COVID-19 outbreaks due to a severe deficiency in B lymphocyte-mediated specific immune response [22].

Further subgroup analysis of lymphoma patients was performed according to the disease diagnosis. The results showed that the immune response ability was in the order of FL, MCL, DLBCL, MZL, TCL, and HL from weak to strong, which was consistent with the treatment characteristics of different types of lymphoma [23–25]. BCL, including FL, MCL, DLBCL, and MZL, requires long-term application of CD20 mAbs and/or B-cell pathway inhibitors, leading to a decrease in humoral immune response ability [10].

The clinical factors that might lead to defective humoral immune response in lymphoma patients were first analyzed. The results confirmed that age, gender, lymphoma staging, disease status, and COVID-19 severity seem to have little impact on immune response. However, vaccination history significantly affected the intensity of humoral immune response, i.e., IgG APR and Ab level. The results demonstrated that vaccination before infection could improve the humoral response to live SARS-CoV-2 in lymphoma patients, which is consistent with previous studies [16, 26]. Thus, as a key aspect for clinical management, protecting vulnerable groups with immune deficiencies, such as lymphoma through vaccines, can reduce the burden on the healthcare system [27, 28]. In addition, our results showed that the use of dexamethasone in the treatment of COVID-19 would affect the Ab level in lymphoma patients. Due to the fact that glucocorticoids interfered with humoral immunity by inhibiting the conversion of B cells to plasma cells, resulting in decreased Ab production [29].

From the viewpoint of therapy-related factors, the times of anti-CD20 and ASCT treatments before infection had adverse effects on the production of anti-SARS-CoV-2 IgG Ab. Previous investigations had reported that the humoral immune response of patients with BCL to SARS-CoV-2 was related to bendamustine and the timing of last anti-CD20 treatment prior to infection [16, 30]. The current study further revealed the impact of the number of anti-CD20 treatments on the humoral response of lymphoma patients when facing SARS-CoV-2 infection. In fact, our results revealed that patients who received anti-CD20 treatment ≥ 6 times exhibited significantly reduced anti-SARS-CoV-2 IgG APR and Ab levels before COVID-19 infection. This indicated that if lymphoma patients were frequently exposed to CD20 mAbs, leading to continuous depletion of B cells, and it would seriously affect their initial immune response to new pathogens. Multifactorial analysis likewise confirmed that the number of anti-CD20 treatment was an independent predictor on APR and Ab levels. The treatment with B-cell-directed therapies led to the depletion of B cells, which might be detrimental to the production of Abs against SARS-CoV-2 in lymphoma patients [10, 31, 32]. Therefore, patients who had been actively treated with CD20 mAbs for a prolonged period might fail to produce protective Abs even after multiple vaccinations and require stronger physical protection against SARS-CoV-2 reinfection [33].

Hematopoietic stem cell transplantation recipients are considered to be at high risk for adverse outcomes after COVID-19 infection due to their immunosuppressive status [34]. Not surprisingly, the results showed that the anti-SARS-CoV-2 IgG APR and Ab levels in lymphoma patients who underwent ASCT prior to COVID-19 infection were significantly lower than those in non-transplant patients. Multifactorial analysis also confirmed that pre-infection ASCT reduced APR. This might be due to the fact that it took a long time for humoral immunity reconstitution after high-dose chemotherapy during ASCT, and the recovery time of peripheral blood B lymphocytes was about three months to over one year [35]. Furthermore, the functional recovery of B cells even took a longer time. This is because the functional recovery of B cells requires the assistance of T cells, which are functionally deficient for a long period after ASCT, thus affecting the functional reconstitution of B cells [36].

Consistent with previous reports [37], the present study likewise confirmed higher absolute B-lymphocyte count in the anti-SARS-CoV-2 IgG positive group than in the negative group in lymphoma patients. Multifactorial analysis found a marginally positive correlation between B-lymphocyte count and anti-SARS-CoV-2 IgG APR. The above results suggested that CD19 + B lymphocyte counts were critical for obtaining anti-SARS-CoV-2 IgG after COVID-19 infection in lymphoma patients. In addition, Bange et al. [38] found that patients with higher number of CD8 + T cells, including those treated with anti-CD20, had improved survival when humoral immunity was deficient. Therefore, CD8 + T cells might contribute to the recovery of COVID-19. However, there were no significant differences in CD4 + T, CD8 + T, and NK cells between the IgG positive and negative groups in the present study. This might be due to the fact that the data in this study on lymphocyte subsets were collected two months after infection, at which the acute phase of viral infection had passed and the cellular immune response was essentially over.

Finally, all lymphoma patients completed a one-year follow-up regarding their clinical outcomes after COVID-19 infection. Although the results indicated that SARS-CoV-2 IgG levels did not relate to clinical outcomes, up to 21.3% of patients in this study had disease progression and nine cases eventually died, due to the interruption of lymphoma treatment caused by COVID-19 infection. Thus, for these patients, poor prognosis due to delayed treatment of lymphoma should be avoided as much as possible.

However, the present study has a few limitations to be improved in future studies. First of all, expect DLBCL, relatively few cases were included in other disease subgroups. Results for those subgroups need to be interpreted with caution and confirmed in a larger cohort. For the influencing factors on Ab level, more detailed hierarchical analyses are still needed, such as the time difference between the last COVID-19 vaccination and the infection, and whether the patients are combined with other infectious diseases or autoimmune diseases.

In summary, the investigation of the humoral immune response ability of lymphoma patients to SARS-CoV-2 has significant clinical and epidemiological significance. The current findings provide strong evidence regarding the reduced ability of producing Ab to SARS-CoV-2 in lymphoma patients. More importantly, the results showed that multiple factors have impacts on the anti-SARS-CoV-2 IgG APR and Ab levels in lymphoma patients, including the vaccination history, the number of anti-CD20 treatments received prior to COVID-19 infection, ASCT therapy before infection, and B-lymphocyte counts. These results may provide references for vaccination strategies and clinical management in lymphoma patients.

### Electronic supplementary material

Below is the link to the electronic supplementary material.

Supplementary Material 1

Supplementary Material 2

## Data Availability

No datasets were generated or analysed during the current study.

## References

[1] Cai J, Deng X, Yang J (2022). Modeling transmission of SARS-CoV-2 Omicron in China. Nat Med.

[2] Pagano L, Salmanton-García J, Marchesi F (2021). COVID-19 infection in adult patients with hematological malignancies: a European Hematology Association Survey (EPICOVIDEHA). J Hematol Oncol.

[3] Maneikis K, Šablauskas K, Ringelevičiūtė U (2021). Immunogenicity of the BNT162b2 COVID-19 mRNA vaccine and early clinical outcomes in patients with haematological malignancies in Lithuania: a national prospective cohort study. Lancet Haematol.

[4] Seow J, Graham C, Merrick B (2020). Longitudinal observation and decline of neutralizing antibody responses in the three months following SARS-CoV-2 infection in humans. Nat Microbiol.

[5] Lumley SF, Wei J, O’Donnell D (2021). The Duration, Dynamics, and determinants of severe Acute Respiratory Syndrome Coronavirus 2 (SARS-CoV-2) antibody responses in Individual Healthcare Workers. Clin Infect Dis.

[6] Wajnberg A, Amanat F, Firpo A (2020). Robust neutralizing antibodies to SARS-CoV-2 infection persist for months. Science.

[7] Dan JM, Mateus J, Kato Y (2020). Immunological memory to SARS-CoV-2 assessed for up to eight months after infection. bioRxiv.

[8] He Z, Ren L, Yang J (2021). Seroprevalence and humoral immune durability of anti-SARS-CoV-2 antibodies in Wuhan, China: a longitudinal, population-level, cross-sectional study. Lancet.

[9] Cattaneo C, Cancelli V, Imberti L (2021). Production and persistence of specific antibodies in COVID-19 patients with hematologic malignancies: role of Rituximab. Blood Cancer J.

[10] Ghione P, Gu JJ, Attwood K (2021). Impaired humoral responses to COVID-19 vaccination in patients with lymphoma receiving B-cell-directed therapies. Blood.

[11] Chang A, Akhtar A, Linderman SL (2022). Humoral responses against SARS-CoV-2 and variants of concern after mRNA vaccines in patients with Non-hodgkin Lymphoma and chronic lymphocytic leukemia. J Clin Oncol.

[12] Cang S, Mukhi N, Wang K (2012). Novel CD20 monoclonal antibodies for lymphoma therapy. J Hematol Oncol.

[13] Tilch MK, Visco C, Kinda S (2022). Outcome of COVID-19 in patients with Mantle Cell Lymphoma-Report from the European MCL Registry. Hemasphere.

[14] Roeker LE, Knorr DA, Thompson MC (2021). COVID-19 vaccine efficacy in patients with chronic lymphocytic leukemia. Leukemia.

[15] Herishanu Y, Avivi I, Aharon A (2021). Efficacy of the BNT162b2 mRNA COVID-19 vaccine in patients with chronic lymphocytic leukemia. Blood.

[16] Liebers N, Speer C, Benning L (2022). Humoral and cellular responses after COVID-19 vaccination in anti-CD20-treated lymphoma patients. Blood.

[17] Qu JM, Wang C, Cao B, on behalf of Chinese Thoracic Society and Chinese Association of Chest Physicians (2020). Guidance for the management of adult patients with coronavirus disease 2019. Chin Med J.

[18] Kong WH, Zhao R, Zhou JB (2020). Serologic Response to SARS-CoV-2 in COVID-19 patients with different severity. Virol Sin.

[19] Xie J, Ding C, Li J (2020). Characteristics of patients with coronavirus disease (COVID-19) confirmed using an IgM-IgG antibody test. J Med Virol.

[20] Turner JS, Kim W, Kalaidina E (2021). SARS-CoV-2 infection induces long-lived bone marrow plasma cells in humans. Nature.

[21] Halliley JL, Tipton CM, Liesveld J (2015). Long-lived plasma cells are contained within the CD19(-)CD38(hi)CD138(+) subset in human bone marrow. Immunity.

[22] Visco C, Marcheselli L, Mina R (2022). A prognostic model for patients with lymphoma and COVID-19: a multicentre cohort study. Blood Adv.

[23] Hoppe RT, Advani RH, Ai WZ (2022). NCCN Guidelines® insights: Hodgkin Lymphoma, Version 2.2022. J Natl Compr Canc Netw.

[24] Zelenetz AD, Gordon LI, Chang JE (2021). NCCN Guidelines® insights: B-Cell Lymphomas, Version 5.2021. J Natl Compr Canc Netw.

[25] Horwitz SM, Ansell S, Ai WZ, Lymphomas T-C (2022). Version 2.2022, NCCN Clinical Practice guidelines in Oncology. J Natl Compr Canc Netw.

[26] Perry C, Luttwak E, Balaban R (2021). Efficacy of the BNT162b2 mRNA COVID-19 vaccine in patients with B-cell non-hodgkin lymphoma. Blood Adv.

[27] Li M, Wang H, Tian L (2022). COVID-19 vaccine development: milestones, lessons and prospects. Signal Transduct Target Ther.

[28] Di Fusco M, Lin J, Vaghela S (2022). COVID-19 vaccine effectiveness among immunocompromised populations: a targeted literature review of real-world studies. Expert Rev Vaccines.

[29] Shimba A, Ikuta K (2020). Control of immunity by glucocorticoids in health and disease. Semin Immunopathol.

[30] Vanni A, Salvati L, Mazzoni A (2023). Bendamustine impairs humoral but not cellular immunity to SARS-CoV-2 vaccination in rituximab-treated B-cell lymphoma-affected patients. Front Immunol.

[31] Ishio T, Tsukamoto S, Yokoyama E, et al. Anti-CD20 antibodies and bendamustine attenuate humoral immunity to COVID-19 vaccination in patients with B-cell non-hodgkin lymphoma. Ann Hematol. 2023;1–11. 10.1007/s00277-023-05204-7 10.1007/s00277-023-05204-7 PMC10089694 37041299

[32] Molica S, Giannarelli D, Lentini M (2022). Efficacy of the BNT162b2 mRNA COVID-19 vaccine in patients with chronic lymphocytic leukemia: a Serologic and Cellular Study. Chemotherapy.

[33] Furlan A, Forner G, Cipriani L (2021). COVID-19 in B Cell-depleted patients after Rituximab: a diagnostic and therapeutic challenge. Front Immunol.

[34] Sharma A, Bhatt NS, St Martin A (2021). Clinical characteristics and outcomes of COVID-19 in haematopoietic stem-cell transplantation recipients: an observational cohort study. Lancet Haematol.

[35] Porrata LF, Litzow MR, Markovic SN. Immune reconstitution after autologous hematopoietic stem cell transplantation. Mayo Clin Proc, 2001. 76(4): 407 – 12. 10.4065/76.4.407 10.4065/76.4.407 11322356

[36] Schlenke P, Sheikhzadeh S, Weber K (2001). Immune reconstitution and production of intracellular cytokines in T lymphocyte populations following autologous peripheral blood stem cell transplantation. Bone Marrow Transplant.

[37] Okamoto A, Fujigaki H, Iriyama C (2022). CD19-positive lymphocyte count is critical for acquisition of anti-SARS-CoV-2 IgG after vaccination in B-cell lymphoma. Blood Adv.

[38] Bange EM, Han NA, Wileyto P (2021). CD8(+) T cells contribute to survival in patients with COVID-19 and hematologic cancer. Nat Med.

